# Supplementary material for: Mechanistic basis of the inhibition of SLC11/NRAMP-mediated metal ion transport by bis-isothiourea substituted compounds
Source: eLife. 2019 Dec 5;8:e51913. doi: 10.7554/eLife.51913 (PMC6917499; doi:10.7554/eLife.51913)
Supplement: Transparent reporting form [file elife-51913-transrepform.docx]

***eLife’s* transparent reporting form**

We encourage authors to provide detailed information *within their submission* to facilitate the interpretation and replication of experiments. Authors can upload supporting documentation to indicate the use of appropriate reporting guidelines for health-related research (see [EQUATOR Network](about:blank)), life science research (see the [BioSharing Information Resource](about:blank)), or the [ARRIVE guidelines](about:blank) for reporting work involving animal research. Where applicable, authors should refer to any relevant reporting standards documents in this form.

If you have any questions, please consult our Journal Policies and/or contact us: [editorial@elifesciences.org](about:blank).

**Sample-size estimation**

- You should state whether an appropriate sample size was computed when the study was being designed
- You should state the statistical method of sample size computation and any required assumptions
- If no explicit power analysis was used, you should describe how you decided what sample (replicate) size (number) to use

Please outline where this information can be found within the submission (e.g., sections or figure legends), or explain why this information doesn’t apply to your submission:

- Iron uptake measurements shown in Figures 1 and Figure 5 were repeated until additional replicates did not substantially alter the average values. The number of biological replicates ranged from 3–39, as indicated in figure legends. Errors are shown as s.d. for the indicated number of replicates in Figure 1B; Figure 1-figure Supplement 2A-G; Figure 1-figure Supplement 3A-D; Figure 5B-C, Figure 5-figure Supplement 1A-E.
- For the proteo-liposome data in Figure 2B, Figure 4D, Figure 4-figure Supplement 2D-F, data from at least 4 technical replicates are shown for a single representative proteo-liposome reconstitution batch. Reconstitutions using different batches of purified protein and synthetic lipid lots resulted in similar data.

**Replicates**

- You should report how often each experiment was performed
- You should include a definition of biological versus technical replication
- The data obtained should be provided and sufficient information should be provided to indicate the number of independent biological and/or technical replicates
- If you encountered any outliers, you should describe how these were handled
- Criteria for exclusion/inclusion of data should be clearly stated
- High-throughput sequence data should be uploaded before submission, with a private link for reviewers provided (these are available from both GEO and ArrayExpress)

Please outline where this information can be found within the submission (e.g., sections or figure legends), or explain why this information doesn’t apply to your submission:

- Number and type of replicates are indicated in the respective figure legends.
- For iron uptake experiments, all replicates are biological. Each experiment was performed in duplicates for transiently transfected cells with data obtained from at least 2 independent transfections or triplicates for stably overexpressed WT hDMT1.
- For proteo-liposome experiments, the technical replicates are measured from different aliquots prepared from a single batch of reconstituted proteo-liposomes.
- For ITC and pH titration experiments, similar results were obtained for at least two experiments from independent protein or compound preparations.
- No obvious outliers related to the behavior of independent cells or protein constructs were encountered or omitted from presented data. Only outliers resulting from technical complications, were discarded.
- Presented data is in accordance with eLife’s regulations and appropriate information regarding the number and type of replicates is provided in the figure legends and methods section.

**Statistical reporting**

- Statistical analysis methods should be described and justified
- Raw data should be presented in figures whenever informative to do so (typically when N per group is less than 10)
- For each experiment, you should identify the statistical tests used, exact values of N, definitions of center, methods of multiple test correction, and dispersion and precision measures (e.g., mean, median, SD, SEM, confidence intervals; and, for the major substantive results, a measure of effect size (e.g., Pearson's r, Cohen's d)
- Report exact p-values wherever possible alongside the summary statistics and 95% confidence intervals. These should be reported for all key questions and not only when the p-value is less than 0.05.

Please outline where this information can be found within the submission (e.g., sections or figure legends), or explain why this information doesn’t apply to your submission:

- X-Ray data collection and refinement statistics were calculated using the programs XDS and Phenix as described in the methods section.
- The sample size, type and number of replicas and the displayed error type is described in each figure legend.
- Raw data used for determination of the IC_50_ values shown in Figure 1B and Table 4 is shown in Figure 1-figure Supplement 2 and Figure 5-figure Supplement E, respectively. Raw data used to determine the kinetic parameters shown in Table 1 is shown in Figure 1-figure Supplement 3, Figure 2B, Figure 2-figure Supplement 1C and in Figure 4-figure Supplement 2A-F.

(For large datasets, or papers with a very large number of statistical tests, you may upload a single table file with tests, Ns, etc., with reference to sections in the manuscript.)

**Group allocation**

- Indicate how samples were allocated into experimental groups (in the case of clinical studies, please specify allocation to treatment method); if randomization was used, please also state if restricted randomization was applied
- Indicate if masking was used during group allocation, data collection and/or data analysis

Please outline where this information can be found within the submission (e.g., sections or figure legends), or explain why this information doesn’t apply to your submission:

N/A

**Additional data files (“source data”)**

- We encourage you to upload relevant additional data files, such as numerical data that are represented as a graph in a figure, or as a summary table
- Where provided, these should be in the most useful format, and they can be uploaded as “Source data” files linked to a main figure or table
- Include model definition files including the full list of parameters used
- Include code used for data analysis (e.g., R, MatLab)
- Avoid stating that data files are “available upon request”

Please indicate the figures or tables for which source data files have been provided:

The coordinates and structure factors of the refined EcoDMT-Br-BIT map have been deposited with the PDB under accession code 6TL2.
